# Risk of HSV-2 Acquisition Among Women with Bacterial Vaginosis: Systematic Review and Meta-Analysis

**DOI:** 10.3390/v18030330

**Published:** 2026-03-07

**Authors:** Taylor N. Whitt, Alexis Heath, D’Atra J. Hill, Douglas K. Brubaker, Christina Farr Zuend

**Affiliations:** 1 Center for Global Health and Diseases, Department of Pathology, School of Medicine, Case Western Reserve University, 10900 Euclid Ave, Cleveland, OH 44106, USA; tnw24@case.edu (T.N.W.); aeh181@case.edu (A.H.);; 2 Blood Heart Lung Immunology Research Center of University Hospitals and Case Western Reserve University, Cleveland, OH 44106, USA

**Keywords:** HSV-2, BV, microbiome

## Abstract

Objective: Bacterial vaginosis is a dysbiosis of the vaginal microbiome, typically characterized by a loss of *Lactobacillus*. *Lactobacillus* plays a crucial role in vaginal immunity and protection against sexually transmitted infections. Herpes simplex virus 2, the primary cause of genital herpes, impacts 13% of people worldwide. We undertook this systematic review and meta-analysis to examine the risk of herpes simplex virus 2 acquisition in women with bacterial vaginosis. Secondarily, we examined the impact of bacterial vaginosis on herpes simplex virus 2 shedding, reactivation, and symptoms. Data sources: We searched PubMed, EMBASE, Cochrane, Web of Science, Google Scholar, and ClinicalTrials.gov for articles published before 1 July 2023 for microbiome and herpes simplex virus type 2. Studies were limited to human subjects and the English language. An updated search was performed in January 2026. This study was registered on PROSPERO (CRD42023439139). Methods of study selection: Studies on non-pregnant, reproductive-aged cisgender women that diagnosed bacterial vaginosis by Amsel Criteria, Nugent Scoring or used molecular techniques, and those that detected herpes simplex virus 2 by serological assay or PCR testing were included. Our search identified 863 results with four publications eligible for inclusion. For our secondary outcomes, 40 results were identified regarding herpes simplex virus 2 shedding, with two publications eligible for inclusion, which did not meet our threshold for meta-analysis. There were 21 results identified for herpes simplex virus 2 reaction and 115 results for herpes simplex virus 2 symptoms, with no articles being eligible for inclusion. Tabulation, integration, and results: Quality assessment was performed following data extraction using the quality assessment scales from the Joanna Briggs Institute. Results were extracted, and the pooled hazard ratio was calculated with 95% confidence interval. A total of 1906 women were included in this analysis, and 255 acquired herpes simplex virus 2. The pooled unadjusted hazard ratios produced an effect size of 1.91, (95% confidence interval 1.4649–2.4980), and a *p*-value of <0.0001, while the pooled adjusted hazard ratios produces an effect size of 1.85, (95% confidence interval of 1.3556–2.5162), and a *p*-value of 0.0001 indicating that bacterial vaginosis is associated with a increased risk of herpes simplex virus 2 acquisition. Conclusions: This systematic review with meta-analysis indicates that bacterial vaginosis is associated with a significantly increased risk (91% unadjusted, 85% adjusted) of herpes simplex virus 2 acquisition, indicating that bacterial vaginosis treatment may reduce herpes simplex virus 2 acquisition. A notable limitation of these findings is the relatively small number of studies eligible for inclusion in this systematic review and meta-analysis.

## 1. Introduction

The vaginal microbiome plays a critical role in gynecologic health. While microbial diversity is optimal in other microbiomes, including the gut or oral cavity, the opposite is true of the vaginal microbiome, where it is estimated that an optimal microbiome is composed of *Lactobacilli* species [1]. *Lactobacilli spp*. are lactic acid and hydrogen peroxide-producing bacteria that contribute to vaginal health by promoting acidic pH and resisting colonization of opportunistic bacteria [2,3]. The dominance of *Lactobacilli* in the vaginal microbiome may also contribute to vaginal health and mucosal immunity through additional mechanisms and interactions that are not yet completely understood.

Bacterial Vaginosis (BV) is a dysbiosis of the vaginal microbiome, characterized by a loss of the optimal *Lactobacillus* dominance and an overgrowth of opportunistic anaerobic and facultative anaerobic microbiota such as *Gardnerella vaginalis, Prevotella,* and *Atopobium vaginae* (now *Fannyhessea vaginae*) [2,3,4,5]. Dysbiosis may be accompanied by symptoms including vaginal discharge, odor, and irritation; however, many cases remain asymptomatic [4]. BV can be diagnosed by Amsel criteria, Nugent scoring or molecular criteria. Amsel criteria are often used clinically and are met by observing three of the following four symptoms: pH above 4.5, thin homogenous discharge, fishy odor after application of 10% potassium solution to vaginal smear, and 20% or greater cells on saline microscopy [6]. Nugent scoring uses Gram staining to identify and quantify bacterial species on the sample. The sample is assigned a score of 0–10, in which 0–3 is considered normal, 4–6 is intermediate, and 7–10 denotes BV [7]. Molecular techniques such as 16S ribosomal RNA (rRNA) sequencing or taxon-specific quantitative PCR are predominantly used in research settings to characterize the bacterial composition of a sample. Molecular BV is defined as a lack of *Lactobacillus* dominance and dominance of facultative or obligate anaerobes, which may not be accompanied by clinical symptoms. The molecular approach allows for the identification and grouping of samples demonstrating similar microbiome composition, which can then be further classified as belonging to specific “community state types” [8].

BV affects up to one-third of women in the United States [9]. While BV can be successfully treated with antibiotics, approximately half will experience recurrence within one year. The prevalence and persistence of BV are concerning, as BV has been associated with several adverse health outcomes. For instance, meta-analyses have revealed an almost two-fold increased risk for pre-term birth in women diagnosed with BV [10] and identified BV diagnosis as a risk factor for HIV acquisition [11]. Additionally, a prospective analysis revealed that BV was associated with an increased risk of acquiring chlamydia, gonorrhea, and trichomonas [12]. Meanwhile, several cross-sectional studies have identified an association between BV and STIs such as human papillomavirus infection [13,14] and herpes simplex virus type 2 [15].

Herpes Simplex Virus type 2 (HSV-2) is a viral infection that causes painful genital ulcerations. Like BV, HSV-2 is associated with an increased risk of HIV acquisition (17). While many cross-sectional and observational studies have identified an association between the presence of BV and HSV-2, little is known about the temporal, comorbid relationship between the two. Notably, a 2015 meta-analysis found that HSV-2 infection leads to an increased risk of BV and suggested that HSV-2 infection is a risk factor for BV [16]. The reverse relationship, whether BV increases the risk of HSV-2 acquisition, has not been fully described. A better understanding of this relationship has the potential to further our understanding of the impact of BV on both mucosal immunity and risk factors for the acquisition of viral STIs.

## 2. Objectives

This systematic review and meta-analysis aim to evaluate the impact of BV on the risk of HSV-2 acquisition. Secondarily, we aim to evaluate the impact of BV on HSV-2 shedding, reactivation, and symptoms.

## 3. Methods

### 3.1. Registration and Protocol

This review was conducted in accordance with the PRISMA guidelines [17], which are designed to improve the transparency, consistency, and thoroughness of reporting in systematic reviews. Additionally, this review and meta-analysis were registered with the International Prospective Register of Systematic Reviews (PROSPERO) database (registration number: CRD42023439139), and the protocol has been published at https://www.crd.york.ac.uk/prospero/display_record.php?RecordID=439139 to promote research transparency, reduce potential bias, and prevent research duplication.

Differences from protocol: Our original protocol, submitted to the PROSPERO database on 23 June 2023, included cross-sectional studies. Upon commencement of this study and further review of previously published meta-analyses, we narrowed our inclusion criteria to include prospective studies only to generate a unique literature review and meta-analysis. These alterations to our protocol were submitted to PROSPERO per their guidelines prior to submission of this article for publication.

### 3.2. Eligibility Criteria

Our review included published studies on cisgender women who were within one year of reproductive age as defined by the World Health Organization (14–50 years of age) and not pregnant. The studies included were prospective observational studies that reported a measure of association between BV and HSV-2. To be included, the studies must determine BV status by Amsel Criteria, Nugent Scoring or molecular techniques, and HSV-2 status by serological assay or PCR testing. Studies that were not available in English, as well as posters and conference abstracts, were excluded.

### 3.3. Sources

Studies were identified by searching the electronic databases PubMed, Web of Science, Embase, CENTRAL, and Google Scholar. There were no restrictions placed on the publication date. Searches of all databases were completed in July 2023. ClinicalTrials.gov was manually searched for relevant trials. An updated search was performed in January 2026.

### 3.4. Search Strategy

Search terms were identified by reviewing relevant literature and utilizing the Medical Subject Headings (MeSH) database, keywords in relevant literature, and subject experts at Case Western Reserve University. Search strings were translated for additional search engines manually. There were no limits applied to searches. See Supplementary Tables S1–S6 for complete search terms.

### 3.5. Study Selection

Identified articles were imported into the bibliographic manager, Zotero (7.0). Duplicates were initially removed automatically using the duplicate feature within Zotero. Any remaining duplicates were tagged during the selection process and manually removed. Two reviewers (T.N.W., D.J.H.) independently reviewed the titles and abstracts of all identified articles to assess for inclusion eligibility. The full article was retrieved for each article that was identified as a candidate for inclusion, and two reviewers (T.N.W., D.J.H.) independently screened the full article for inclusion. Inclusion decisions were recorded by labeling each article with an “include” or “exclude” tag within Zotero. In cases in which full article access was unavailable due to institutional access, the full article was retrieved via interlibrary loan. In cases of discordant inclusion decisions between the two reviewers, a third reviewer (C.F.Z.) reviewed the full article and made a final inclusion decision.

### 3.6. Data Extraction

One author (T.N.W.) independently extracted data from the included studies into Excel spreadsheets. A second author (D.J.H.) independently reviewed articles to confirm that the data extracted was accurate. Any discrepancies in data extraction were resolved by a third reviewer (C.F.Z.).

### 3.7. Data Items

The data items extracted that related to outcomes of interest were the measure of association (HR, OR, RR, Likelihood Ratio) between BV and incident HSV-2, including confidence intervals and p-scores, and confounders included in adjusted models. Additional data items extracted included study design, study country, study size, participant inclusion criteria, diagnostic method of BV, diagnostic method of HSV-2, method of characterizing the vaginal microbiome (if any), the incidence of BV, the prevalence of BV, the incidence of HSV-2, and the prevalence of HSV-2. We also extracted information related to funding sources. All data was extracted manually.

### 3.8. Assessment of Risk of Bias

Risk of bias was assessed using appropriate quality assessment scales from the Joanna Briggs Institute (JBI) [18]. Each study included was assessed using the JBI Critical Appraisal Tool for Cohort Studies or the JBI Critical Appraisal Tool for Cross-Sectional Studies, as applicable. The tools were applied to each study by two reviewers (T.N.W., C.F.Z.) independently to assess for risk of bias and determine inclusion eligibility. A third reviewer (D.J.H.) was available to apply the tool in the case of discrepancies.

### 3.9. Effect Measures

To investigate the association between BV infection and HSV-2 acquisition, we analyzed the hazard ratio (HR) values and 95% confidence intervals of HSV-2 infection in patients with BV as reported by individual studies.

### 3.10. Data Analysis

Meta-analyses were conducted in R (R version 4.4.0, RStudio Version 2024.04.2+764) using the *metagen* function from the R package *meta*. This function uses an inverse variance method, requiring treatment estimates (TE) and standard errors (SE) as inputs. Considering between-study heterogeneity, a random effects meta-analysis was performed using the hazard ratio values and confidence intervals provided by each study. SE was approximated by *metagen* from the provided confidence intervals. Two sets of meta-analyses were conducted based on either the adjusted or unadjusted test values provided by all studies, creating two distinct meta-analysis results: Unadjusted and Adjusted Meta Analysis.

### 3.11. Reporting Bias Assessment

For each result, the random effects model produced a pooled effect size in the form of a hazard ratio, along with its 95% confidence interval and *p*-value. Funnel plots and Egger’s test were run to determine potential publication bias or small study effects. Sensitivity analyses were also performed to quantify how the pooled effect size would change if a given study were removed from the meta-analysis.

## 4. Results

The results of this systematic review and meta-analysis are presented according to PRISMA guidelines.

### 4.1. Study Selection

#### 4.1.1. Primary Outcome

Our initial search of PubMed, EMBASE, Cochrane, Web of Science, and Google Scholar yielded 863 results, 377 of which were duplicates and removed by automation tools or manually prior to screening. A manual search of ClinicalTrials.gov did not identify any additional results. In total, 486 individual studies were screened for relevance. Upon initial screening of abstracts and titles, 436 articles were excluded for non-relevance to our study question. Thus, 50 articles were retrieved for full-text review. Ten studies were excluded on the basis that they were conference abstracts, and additional searches did not yield full, published studies. Sixteen studies were excluded due to study type; most of them were cross-sectional studies, while two were meta-analyses. Five studies were excluded based on directionality, as they were examining incident BV, while five additional studies were excluded for failure to report incident HSV-2. Three studies were excluded because they failed to report any data on HSV-2 and BV co-infection. Upon full-text review, two studies were found not to be relevant to our investigation, as one was an animal study, while the second was analyzing ex vivo cultures. Two studies included pregnant women and therefore did not meet our population inclusion criteria, while two studies did not meet our diagnostic criteria for BV or HSV-2. Finally, one study that appeared to meet the inclusion criteria was excluded due to the unavailability of data and our inability to contact the corresponding author.

**Figure 1:**
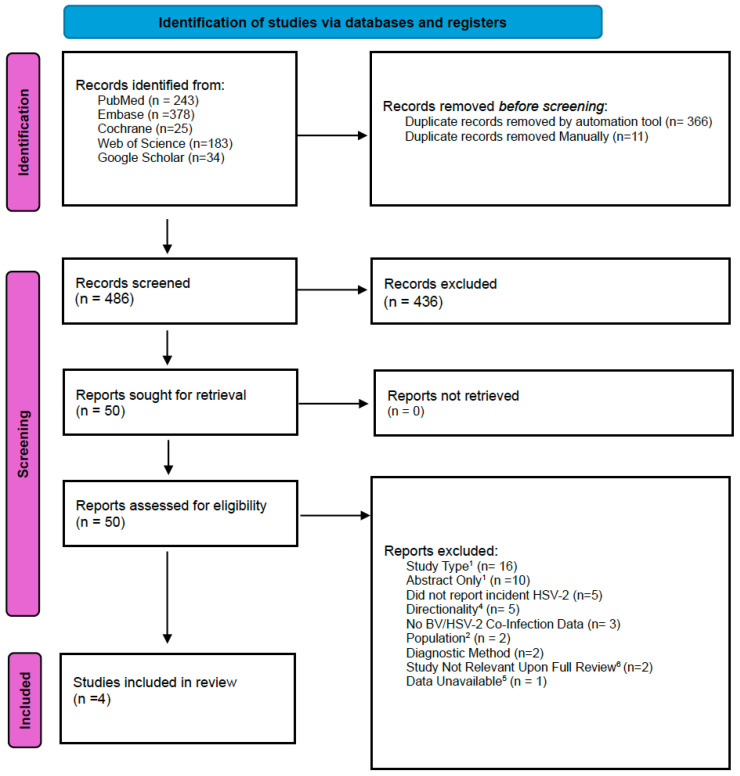
PRISMA flow chart. A visualization of the identification, screening, and eligibility assessment of included studies for the primary outcome. ^1^ 1 Meta-analysis, 15 cross-sectional ^1^ Additional search for full published study conducted before excluding ^4^ Reported incident BV rather than incident HSV-2 ^2^ Pregnant women included in analysis ^6^ One animal study, one analysis of ex vivo mucosal cultures ^5^ Unable to contact authors.

Ultimately, four studies met all inclusion criteria and were selected for meta-analysis. (Figure 1) An updated search was performed in January 2026, which identified one additional article for full-text review. Upon full-text review, this study did not meet our population and diagnostic inclusion criteria and was excluded.

#### 4.1.2. Secondary Outcomes

In addition to the primary outcome of interest, this study conducted searches on three additional outcomes: the association between BV and HSV-2 reactivation, the association between BV and the occurrence of HSV-2 symptoms, and the association between BV and HSV-2 viral shedding. These searches were also conducted on PubMed, EMBASE, Cochrane, Web of Science, and Google Scholar.

Our search regarding the association between BV and HSV-2 viral shedding resulted in 40 articles; 19 of which were duplicates, yielding 21 unique studies. Upon review of the title and abstract, 17 studies were excluded for relevance. Four studies were retrieved for full-text review. Upon review of the full texts, one article was excluded based on directionality, as it examined BV acquisition and resistance to treatment in the setting of HSV-2. Additionally, one article was excluded for failing to report any association between BV and HSV-2 shedding. Ultimately, two studies met our inclusion criteria. This did not meet the threshold of four studies needed to be included in the meta-analysis. (Supplementary Figure S1).

Our initial search regarding the association between BV and HSV-2 reactivation yielded 21 results, 8 of which were duplicates, yielding 13 unique studies. Upon title and abstract review, all articles were excluded for relevance. See supplemental materials for PRISMA flow diagram.

Finally, our search regarding the association between BV and the occurrence of HSV-2 symptoms yielded 115 results, 50 of which were duplicates, yielding 65 individual articles. Upon review of the title and abstract, 62 articles were excluded for relevance. Three full articles were retrieved for full review. Upon full review, all three articles were excluded for failing to report an association between BV and the occurrence of HSV-2 symptoms.

A secondary search was performed in January 2026, and no additional articles for any of the secondary outcomes were identified for full-text review.

### 4.2. Assessment of Risk of Bias

#### 4.2.1. Primary Outcome

The studies in this systematic review and meta-analysis were found to be of moderate or high quality using the JBI Critical Appraisal tool for cohort studies. Most of the studies met at least 10 of the 11 criteria on the appraisal checklist, and all of them met at least 9 criteria. Each study was determined to be of an acceptable quality for inclusion in the overall appraisal by each reviewer. Additionally, all four studies were considered to have a low degree of bias related to funding. A review of financial disclosures revealed that all studies reported their funding sources, and none reported a conflict of interest. (Supplementary Table S7).

#### 4.2.2. Secondary Outcome

The studies included for the secondary outcome were evaluated using the JBI Critical Appraisal Tool for Cross-Sectional Studies. Both studies met all 8 criteria on the appraisal checklist and were determined to be of an acceptable quality for inclusion by each reviewer. Both studies were considered to have a low degree of bias related to funding. A review of financial disclosures revealed that both studies disclosed funding sources and reported no conflicts of interest for all authors. (Supplementary Table S8).

### 4.3. Results of Individual Studies

#### 4.3.1. Primary Outcome

Each of the four included articles was a prospective cohort study. These four studies were conducted between the years 1992 and 2006, and published between the years 2003 and 2009. Three of the four studies were conducted in the United States, while one was conducted in Kenya. The aim of three of the studies was to identify risk factors for incident HSV-2 infection, while one study explicitly aimed to examine BV as a risk factor for incident HSV-2. Participants in all four studies were HSV-2 seronegative at the start of the study, with HSV-2 acquisition being defined as conversion to HSV-2 seropositive on either IgG assay or serological ELISA testing. The length of follow-up in these studies ranged from 6 months to 4.5 years, with follow-up intervals occurring monthly, quarterly, or biannually. Participants for all four studies were recruited from those attending STI clinics. One of the four studies [19] recruited participants who self-reported exchanging sex for money or attending an STI clinic intended for sex workers. In total, 1906 participants were included across all four studies. Of these participants, 255 seroconverted to HSV-2 positive during the duration of the study. Additionally, all four studies found a positive correlation between BV and the acquisition of HSV-2 infection, indicating that BV increased the risk of acquiring HSV-2. All four of the studies reported a hazard ratio, with an adjusted hazard ratio ranging from 1.56 (95% CI = 0.96–2.55, *p* = 0.07) to 2.4 (95% CI = 1.1–5.6, *p* = 0.05) [19,20,21,22] (Table 1).

#### 4.3.2. Secondary Outcome

Both studies included were observational cross-sectional studies. Baisley et al. [23] conducted a baseline analysis of a cohort of women enrolled in a randomized, double-blind, placebo-controlled trial of acyclovir. The aim of this study was to determine the prevalence of, and risk factors for, BV among HSV-2 seropositive women at enrollment of the trial. The researchers recruited HSV-2 seropositive women aged 16–35 years old, who were working in bars and guesthouses in the Lake Victoria region of Tanzania. In total, 1304 women were enrolled in the study. Of these participants, 807 had BV and 34 had detectable genital HSV-2 shedding. Multivariable analysis found that detectable HSV-2 shedding was not significantly associated with BV (aOR 0.93; 95% CI (0.60–1.46), *p* = 0.77).

The aim of Cherpes et al. [24] was to investigate the effects of vaginal coinfections and hormonal contraceptive use on genital tract HSV-2 shedding in women. This study recruited HSV-2 seropositive women aged 18–30 years old from three clinics in the Philadelphia, Pennsylvania area. To be eligible, these women could not be using anti-viral medication. After enrollment, participants were followed up at 4-month intervals for one year. 330 HSV-2 seropositive women were included in this study. In total, participants accrued 956 study visits, and detectable genital HSV-2 shedding was identified in 88 samples. HSV-2 shedding was observed more often in women with BV. In contrast to the Baisley et al. study, multivariate analysis of this data identified BV as an independent risk factor for HSV-2 shedding (aOR 2.3, 95% (1.3–4.0), *p* = 0.003). (Supplementary Table S9).

### 4.4. Meta-Analysis

**Figure 2:**
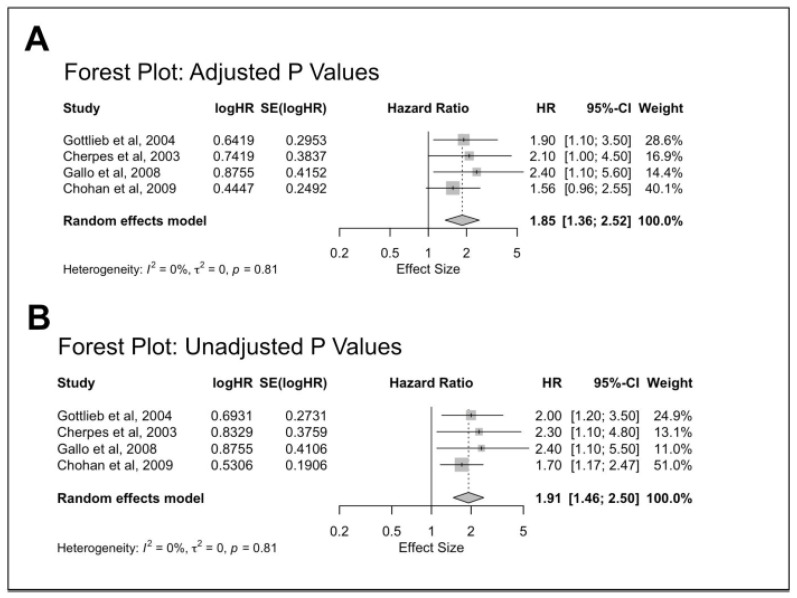
(**A**) Forest plot of cross-sectional associations between herpes simplex virus type 2 (HSV-2) infection and bacterial vaginosis (BV) using Adjusted *p* Values. (**B**) Forest plot of cross-sectional associations between herpes simplex virus type 2 (HSV-2) infection and bacterial vaginosis (BV) using Unadjusted *p* Values. Cherpes et al. 2003 [20], Chohan et al. 2009 [19], Gallo et al. 2008 [21], Gottlieb et al. 2004 [22].

All four included studies reported a positive association between BV diagnosis and acquisition of HSV-2, although the adjusted *p-values* were not statistically significant (*p* = 0.05–0.07). The unadjusted hazard ratio values produced a pooled effect size of 1.91, a 95% confidence interval of [1.4649; 2.4980], and a *p*-value of <0.0001 (Figure 2A). The heterogeneity test was insignificant with a *p*-value of 0.8120 (Figure 2A). Chohan et al. (2009) [19] contributed most heavily to the overall heterogeneity and had the most influence on the overall result (Table 2). A funnel plot was generated to check for potential bias in publication or small study effects (Supplementary Table S10). Slight asymmetry indicated to run Egger’s test. The results of Egger’s test gave a z-value of 0.6506 and a *p*-value of 0.5153, suggesting that the effect sizes from the included studies are likely not systematically biased due to publication bias or small study effects. Sensitivity analysis indicated that the pooled effect size would not significantly differ from the originally calculated pooled effect size if any study were removed (Table 3).

When considering the adjusted hazard ratio values, they produced a pooled effect size of 1.85, a 95% confidence interval of [1.3556; 2.5162], and a *p*-value of 0.0001 (Figure 2B). The heterogeneity test was insignificant with a *p*-value of 0.8065 (Figure 2B). Chohan et al. (2009) [19] contributed most heavily to the overall heterogeneity and had the most influence on the overall result (Table 2). A funnel plot was generated to check for potential publication bias or small study effects (Supplementary Table S10). Slight asymmetry indicated to run Egger’s test. The results of Egger’s test gave a z-value of 0.6149 and a *p*-value of 0.5386, suggesting that the effect sizes from the included studies are likely not systematically biased due to publication bias or small study effects. Sensitivity analysis indicated that the pooled effect size would not significantly differ from the originally calculated pooled effect size if any study were removed (Table 3).

Thus, considering unadjusted or adjusted pooled estimates indicated there was a 91% (unadjusted) or 85% (adjusted) increased risk for HSV-2 acquisition among participants clinically diagnosed with BV.

## 5. Discussion

### 5.1. Principal Findings

In this systematic review and meta-analysis, BV was significantly associated with an increased risk of HSV-2 acquisition in women 14–50 years of age. This association persisted regardless of subset analysis and across all sensitivity analyses, and was maintained when using both unadjusted and adjusted data. These results support a temporal relationship between BV and HSV-2 and suggest that vaginal dysbiosis is a significant risk factor for HSV-2 acquisition.

While two studies were included in a systematic review of the association between BV and HSV-2 viral shedding, they did not meet our threshold for conducting a meta-analysis. The two studies reported conflicting findings regarding BV as a risk factor for HSV-2 viral shedding. Therefore, we were unable to come to any conclusions on the impact of BV on the occurrence of HSV-2 viral shedding.

### 5.2. Comparison with Existing Literature

Many studies have reported an association between BV and acquisition of STIs, including HIV, chlamydia, gonorrhea, and HPV [12,13,14,15,16]. While many cross-sectional studies have reported data on BV and HSV-2 co-infection, we found very few prospective studies exist that address whether BV itself is associated with an increased risk of HSV-2 acquisition. These findings complement those of a 2015 systematic review and meta-analysis of cross-sectional studies, which identified a significant association between HSV-2 infection and risk of development of BV [18].

### 5.3. Strengths and Limitations

Strengths of this review included our use of a variety of search databases, including PubMed, EMBASE, Cochrane, Web of Science, Google Scholar, and ClinicalTrials.gov. This ensured that we captured as many eligible studies as possible. Additionally, we followed PRISMA guidelines to conduct a literature review and meta-analysis, which was transparent and reproducible.

An important limitation of this meta-analysis is that all included studies relied on convenience sampling of patients who were attending STI clinics or self-identified as sex workers. The incidence of HSV-2 may be inflated in this population, as many were likely presenting to clinics with gynecological symptoms or complaints and, therefore, do not reflect the incidence of HSV-2 in the general population. Additionally, all the included studies utilized clinical Amsel criteria or Nugent scoring to diagnose BV, with no studies undertaking bacterial sequencing. This is likely a reflection of the relative age of the included studies, all of which were published between 2003 and 2009. Performing vaginal microbiome sequencing can detect asymptomatic BV, which could address whether similar results are observed in asymptomatic participants, and may be better able to characterize the influence of specific microorganism composition on vaginal mucosal immunity and inflammation. Given that asymptomatic women can have non-*Lactobacillus* dominant microbiomes [1], and that lack of *Lactobacillus* in the vaginal microbiome, independent of clinical BV diagnosis, is associated with increased risk of acquisition of other STIs [11,12,13,14,15], the lack of papers addressing molecular BV and HSV-2 acquisition is a critical gap in the field.

### 5.4. Conclusions and Clinical Implications

The findings of this meta-analysis demonstrate that BV is associated with increased risk of HSV-2 acquisition in women between 14 and 50 years of age and are aligned with the hypothesis that a lack of *Lactobacillus* dominance in the cervicovaginal environment alters mucosal immunity and inflammation in a way that alters susceptibility to infection by viral pathogens. Indeed, recent in vitro and in vivo studies have investigated the impact of specific members of the vaginal microbiome on HSV-2 infection and have identified decreased vaginal epithelial barrier integrity, activation of interferon signaling pathways, and viral neutralization as potential mechanisms linking the composition of the vaginal microbiome to HSV susceptibility [25,26,27]. Overall, the findings of this meta-analysis could have important implications for HSV-2 prevention through treatment of BV and optimization of the vaginal microbiome. Future clinical research should investigate the impact of BV on HSV-2 reactivation and viral shedding, as well as determine if asymptomatic BV impacts HSV-2 acquisition. Additionally, an investigation into the impact of BV treatment on HSV-2 susceptibility is warranted. Further basic science research must be conducted to characterize the mechanisms by which microorganisms protect against viral infection and alter the mucosal immunity of the vagina.

## Figures and Tables

**Table 1:** Summary of Studies of the Association between BV and HSV-2 Acquisition.

Author	Country	Study Type	Aim	Study Population	Method of BV Dianosis	Participants	Measure of Effect	Estimate (95% CI)	Risk of Bias
Cherpes et al. (2003) [20]	USA	Prospective Cohort	Determine whether the presence of BV is among the risk factors associated with increased rates of HSV-2	HSV-2 seronegative sexually active women (18–30 y/o) attending three healthcare clinics between 1998–2000.	Nugent Scoring	670	HR	2.1 (1.0–4.5)	Low
Chohan et al. (2009) [19]	Kenya	Prospective Cohort	Determine incidence and risk factors for HSV-2 acquisition	HIV -seronegative, HSV-2-seronegative women attending municipal sex worker clinic between 1993–2006.	Nugent Scoring	297	HR	1.56 (0.98–2.55)	Low
Gallo et al. (2008) [21]	USA	Prospective Cohort	To estimate the incidence of HSV-2 and identify risk factors for its acquisition	HSV-2-seronegative Women (18–25 y/o), attending a public STD t between 1992–1995.	Amsel Criteria	293	HR	2.4 (1.1–6.5)	Low
Gottleib et al. (2004) [22]	USA	Prospective Cohort	Determine incidence and incident predictors of HSV-2 infection.	HSV-2 seronegative women who presented for STD examinations at public STD clinics between 1993–1996.	Amsel Criteria	646	HR	1.9 (1.1–3.5)	Low

**Table 2:** Heterogeneity Test Results for both sets of meta-analyses. Each study’s influence on the overall result and its contribution to overall heterogeneity are noted.

Heterogeneity Test Results			
Adjusted or Unadjusted *p* Values	Study Name	Influence on Overall Result (Standardized Squared Difference)	Contribution to Overall Heterogeneity (Cochran’s Q)
Adjusted	Cherpes et al., 2003 [20]	0.11	0.023
	Chohan et al., 2009 [19]	0.46	0.31
	Gallo et al., 2008 [21]	0.40	0.067
	Gottlieb et al., 2004 [22]	0.0092	0.0037
Unadjusted	Cherpes et al., 2003 [20]	0.24	0.036
	Chohan et al., 2009 [19]	0.38	0.4
	Gallo et al., 2008 [21]	0.31	0.038
	Gottlieb et al., 2004 [22]	0.027	0.009

**Table 3:** Influence Analysis of Cross-Sectional Studies showing the estimated effect size and 95% confidence interval if a named study is omitted.

Influence Analysis of Cross-Sectional Studies			
Adjusted or Unadjusted *p* Values	Name of Study Omitted	Effect Size Estimates	95% Confidence Interval
Adjusted	Cherpes et al., 2003 [20]	1.8	[0.85, 2.7]
	Chohan et al., 2009 [19]	2.1	[0.90, 3.2]
	Gallo et al., 2008 [21]	1.8	[0.84, 2.7]
	Gottlieb et al., 2004 [22]	1.8	[0.77, 2.9]
Unadjusted	Cherpes et al., 2003 [20]	1.9	[1.1, 2.7]
	Chohan et al., 2009 [19]	2.2	[1.1, 3.2]
	Gallo et al., 2008 [21]	1.9	[1.1, 2.7]
	Gottlieb et al., 2004 [22]	1.9	[1.0, 2.8]

## Supplementary Materials

The following supporting information can be downloaded at: https://www.mdpi.com/article/10.3390/v18030330/s1, Figure S1: Flow of identification of studies for inclusion in secondary outcome, HSV-2 shedding; Table S1: Primary outcome search terms; Table S2: PubMed search strings; Table S3: Main outcome search strings; Table S4: Reactivation search strings; Table S5: Symptoms search strings; Table S6: Viral shedding search strings; Table S7: Quality assessment of studies included in primary outcome using JBI critical appraisal tool for cohort studies; Table S8: Quality assessment of studies included in secondary outcome using JBI critical appraisal tool for cross-sectional studies; Table S9: Summary of studies on the association between BV and HSV-2 viral shedding; Table S10: Funnel plot results for both sets of meta-analyses.
