# Supplementary material for: Risk of HSV-2 Acquisition Among Women with Bacterial Vaginosis: Systematic Review and Meta-Analysis
Source: Viruses. 2026 Mar 7;18(3):330. doi: 10.3390/v18030330 (PMC13030548; doi:10.3390/v18030330)
Supplement: Supplementary file 1 [file viruses-18-00330-s001.zip › viruses-4153852-supplementary.pdf]

Supplementary Materials:
Supplementary Figure S1. Flow of identification of studies for inclusion in secondary outcome, HSV-2 Shedding

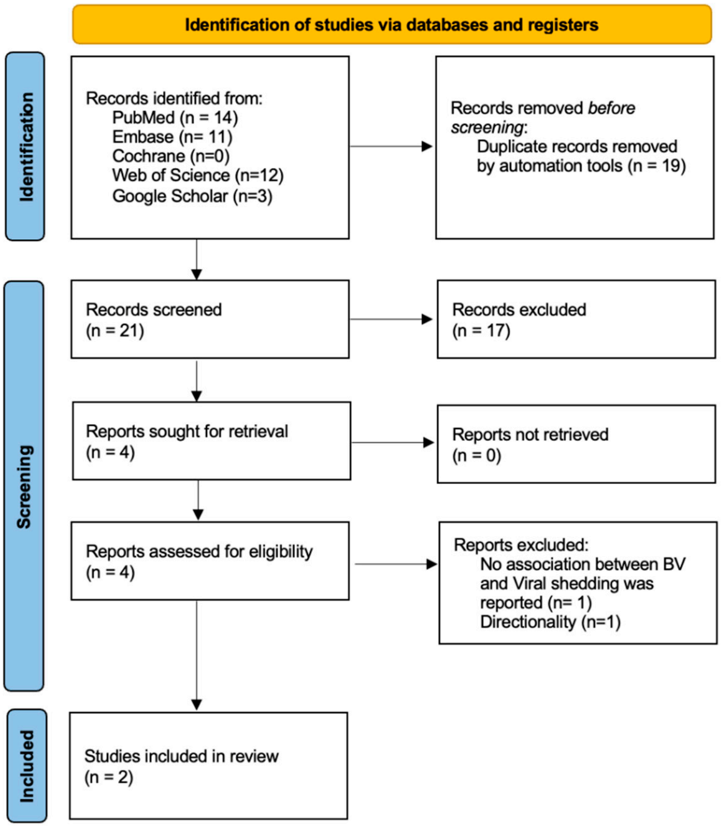

Supplementary Tables

"Primary Outcome Search Strategy"

Supplementary Table S1. Search Terms

Exposure	Outcome	Additional Outcomes of Interest
"Microbiome" OR	"Herpes Simplex Virus Type 2" OR	"Reactivation" OR
"Microbiome" OR	"Herpes Simplex Virus 2" OR	"Viral Shedding" OR
"Vaginal Microbio*" OR	"HSV-2" OR	"Symptom*"

“Female Reproductive Tract Microbio*” OR	“Genital Herpes”	
“Bacterial Vagin*” OR		
“Vaginal Dysbiosis” OR		
“Vaginitis”		

Supplementary Table S2. PubMed Search Strings

**Outcome**	**Search Strings**
HSV-2 Infection	("Microbiota"[MeSH Terms] OR "vaginosis, bacterial"[MeSH Terms] OR "Microbiota"[Title/Abstract] OR "microbiome"[Title/Abstract] OR "vaginal microbio*"[Title/Abstract] OR "bacterial vagin*"[Title/Abstract] OR "female reproductive tract microbio*"[Title/Abstract] OR "vaginal dysbiosis"[Title/Abstract] OR "vaginitis"[Title/Abstract]) AND ("herpesvirus 2, human"[MeSH Terms] OR "herpes simplex virus type 2"[Title/Abstract] OR "herpes simplex virus 2"[Title/Abstract] OR "HSV-2"[Title/Abstract] OR "genital herpes"[Title/Abstract])
HSV-2 Reactivation	… AND (“reactive*”)[1]
Presence of HSV-2 Symptoms	… AND (“symptom*”)[1]
HSV-2 Viral Shedding	… AND (“viral shedding”)[1]

[1]Note that these terms were full text searches, not restricted to the title, abstract, or any other portion of the manuscript

Supplementary Table S3. Main Outcome Search Strings

Database	Search Strings

PubMed	("Microbiota"[MeSH Terms] OR "vaginosis, bacterial"[MeSH Terms] OR "Microbiota"[Title/Abstract] OR "microbiome"[Title/Abstract] OR "vaginal microbio*"[Title/Abstract] OR "bacterial vagin*"[Title/Abstract] OR "female reproductive tract microbio*"[Title/Abstract] OR "vaginal dysbiosis"[Title/Abstract] OR "vaginitis"[Title/Abstract]) AND ("herpesvirus 2, human"[MeSH Terms] OR "herpes simplex virus type 2"[Title/Abstract] OR "herpes simplex virus 2"[Title/Abstract] OR "HSV-2"[Title/Abstract] OR "genital herpes"[Title/Abstract])
Cochrane	([mh Microbiota] OR [mh "vaginosis, bacterial"] OR Microbiota:ti,ab OR microbiome:ti,ab OR ("vaginal" NEXT microbio*):ti,ab OR ("bacterial" NEXT vagin*):ti,ab OR ("female reproductive tract" NEXT microbio*):ti,ab OR "vaginal dysbiosis":ti,ab OR vaginitis:ti,ab) AND ([mh "herpesvirus 2, human"] OR "herpes simplex virus type 2":ti,ab OR "herpes simplex virus 2":ti,ab OR HSV-2:ti,ab OR "genital herpes":ti,ab)
Web of Science	(Microbiota:ti,ab OR microbiome:ti,ab OR 'vaginal microbio*':ti,ab OR 'bacterial vagin*':ti,ab OR 'female reproductive tract microbio*':ti,ab OR 'vaginal dysbiosis':ti,ab OR vaginitis:ti,ab) AND ('herpesvirus 2, human'/exp OR 'herpes simplex virus type 2':ti,ab OR 'herpes simplex virus 2':ti,ab OR HSV-2:ti,ab OR 'genital herpes':ti,ab)
Google Scholar	allintitle: ("bacterial vaginosis" OR "vaginal microbiome" OR "vaginal dysbiosis" OR "Vaginal microbiota" OR "female reproductive tract microbiome" OR "female reproductive tract microbiota" OR) AND ("Herpes Simplex Virus type 2" OR "Herpes simplex virus 2" OR "HSV-2" OR genital herpes")
Embase	((bacterial AND vagin*:ti,ab) OR microbio*:ab,ti OR 'vaginal dysbiosis':ab,ti OR 'vaginitis':ti,ab OR (vaginal AND microbi*:ti,ab) OR (female AND reproductive AND tract AND microbio*:ab,ti)) AND ('herpes simplex virus 2':ab,ti OR 'hsv 2':ab,ti OR 'genital herpes':ab,ti OR 'herpes virus infection':ab,ti)

"Additional Outcomes Search Strings"

Supplementary Table S4. Reactivation

**Database**	**Search String**
PubMed	("Microbiota"[MeSH Terms] OR "vaginosis, bacterial"[MeSH Terms] OR "Microbiota"[Title/Abstract] OR "microbiome"[Title/Abstract] OR "vaginal microbio*"[Title/Abstract] OR "bacterial vagin*"[Title/Abstract] OR "female reproductive tract microbio*"[Title/Abstract] OR "vaginal dysbiosis"[Title/Abstract] OR "vaginitis"[Title/Abstract]) AND ("herpesvirus 2, human"[MeSH Terms] OR "herpes simplex virus type 2"[Title/Abstract] OR "herpes simplex virus 2"[Title/Abstract] OR "HSV-2"[Title/Abstract] OR "genital herpes"[Title/Abstract]) AND "reactiv*"[All Fields]

EMBASE	(microbiota:ti,ab OR microbiome:ti,ab OR 'vaginal microbio*':ti,ab OR 'bacterial vagin*':ti,ab OR 'female reproductive tract microbio*':ti,ab OR 'vaginal dysbiosis':ti,ab OR vaginitis:ti,ab) AND ('herpesvirus 2, human'/exp OR 'herpes simplex virus type 2':ti,ab OR 'herpes simplex virus 2':ti,ab OR 'hsv 2':ti,ab OR 'genital herpes':ti,ab) AND (‘reactivation’)
Cochrane	([mh Microbiota] OR [mh "vaginosis, bacterial"] OR Microbiota:ti,ab OR microbiome:ti,ab OR ("vaginal" NEXT microbio*):ti,ab OR ("bacterial" NEXT vagin*):ti,ab OR ("female reproductive tract" NEXT microbio*):ti,ab OR "vaginal dysbiosis":ti,ab OR vaginitis:ti,ab) AND ([mh "herpesvirus 2, human"] OR "herpes simplex virus type 2":ti,ab OR "herpes simplex virus 2":ti,ab OR HSV-2:ti,ab OR "genital herpes":ti,ab) AND reactiv*
Web of Science	(Microbiota:ti,ab OR microbiome:ti,ab OR 'vaginal microbio*':ti,ab OR 'bacterial vagin*':ti,ab OR 'female reproductive tract microbio*':ti,ab OR 'vaginal dysbiosis':ti,ab OR vaginitis:ti,ab) AND ('herpesvirus 2, human'/exp OR 'herpes simplex virus type 2':ti,ab OR 'herpes simplex virus 2':ti,ab OR HSV-2:ti,ab OR 'genital herpes':ti,ab) AND all=Reactivat*
Google Scholar	allintitle:("bacterial vaginosis" OR "vaginal microbiome" OR "vaginal dysbiosis" OR "Vaginal microbiota" OR "female reproductive tract microbiome" OR "female reproductive tract microbiota" OR) AND ("Herpes Simplex Virus type 2" OR "Herpes simplex virus 2” OR “HSV-2” OR genital herpes”) AND (“reactivation” OR “reactivate”)

Supplementary Table S5. Symptoms

Database	Search String
PubMed	("Microbiota"[MeSH Terms] OR "vaginosis, bacterial"[MeSH Terms] OR "Microbiota"[Title/Abstract] OR "microbiome"[Title/Abstract] OR "vaginal microbio*"[Title/Abstract] OR "bacterial vagin*"[Title/Abstract] OR "female reproductive tract microbio*"[Title/Abstract] OR "vaginal dysbiosis"[Title/Abstract] OR "vaginitis"[Title/Abstract]) AND ("herpesvirus 2, human"[MeSH Terms] OR "herpes simplex virus type 2"[Title/Abstract] OR "herpes simplex virus 2"[Title/Abstract] OR "HSV-2"[Title/Abstract] OR "genital herpes"[Title/Abstract]) AND ("virus shedding"[MeSH Terms] OR ("virus"[All Fields] AND "shedding"[All Fields]) OR "virus shedding"[All Fields] OR ("viral"[All Fields] AND "shedding"[All Fields]) OR "symptom*"[All Fields])
EMBASE	(microbiota:ti,ab OR microbiome:ti,ab OR 'vaginal microbio*':ti,ab OR 'bacterial vagin*':ti,ab OR 'female reproductive tract microbio*':ti,ab OR 'vaginal dysbiosis':ti,ab OR vaginitis:ti,ab) AND ('herpesvirus 2, human'/exp OR 'herpes

	simplex virus type 2':ti,ab OR 'herpes simplex virus 2':ti,ab OR 'hsv 2':ti,ab OR 'genital herpes':ti,ab)AND ('virus shedding'/exp OR "virus shedding")
Cochrane	([mh Microbiota] OR [mh "vaginosis, bacterial"] OR Microbiota:ti,ab OR microbiome:ti,ab OR ("vaginal" NEXT microbio*):ti,ab OR ("bacterial" NEXT vagin*):ti,ab OR ("female reproductive tract" NEXT microbio*):ti,ab OR "vaginal dysbiosis":ti,ab OR vaginitis:ti,ab) AND ([mh "herpesvirus 2, human"] OR "herpes simplex virus type 2":ti,ab OR "herpes simplex virus 2":ti,ab OR HSV-2:ti,ab OR "genital herpes":ti,ab) AND "viral shedding"
Web of Science	(Microbiota:ti,ab OR microbiome:ti,ab OR 'vaginal microbio*':ti,ab OR 'bacterial vagin*':ti,ab OR 'female reproductive tract microbio*':ti,ab OR 'vaginal dysbiosis':ti,ab OR vaginitis:ti,ab) AND ('herpesvirus 2, human'/exp OR 'herpes simplex virus type 2':ti,ab OR 'herpes simplex virus 2':ti,ab OR HSV-2:ti,ab OR 'genital herpes':ti,ab) AND all=shedding
Google Scholar	allintitle: Herpes simplex virus 2 AND ("bacterial vaginosis" OR "vaginal microbiome" OR "vaginal dysbiosis" OR "Vaginal microbiota" OR "female reproductive tract microbiome") AND ("shedding" OR "shed")

Supplementary Table S6. Viral Shedding

**Database**	**Search String**
PubMed	("Microbiota"[MeSH Terms] OR "vaginosis, bacterial"[MeSH Terms] OR "Microbiota"[Title/Abstract] OR "microbiome"[Title/Abstract] OR "vaginal microbio*"[Title/Abstract] OR "bacterial vagin*"[Title/Abstract] OR "female reproductive tract microbio*"[Title/Abstract] OR "vaginal dysbiosis"[Title/Abstract] OR "vaginitis"[Title/Abstract]) AND ("herpesvirus 2, human"[MeSH Terms] OR "herpes simplex virus type 2"[Title/Abstract] OR "herpes simplex virus 2"[Title/Abstract] OR "HSV-2"[Title/Abstract] OR "genital herpes"[Title/Abstract]) AND "symptom*"[All Fields]
EMBASE	(microbiota:ti,ab OR microbiome:ti,ab OR 'vaginal microbio*':ti,ab OR 'bacterial vagin*':ti,ab OR 'female reproductive tract microbio*':ti,ab OR 'vaginal dysbiosis':ti,ab OR vaginitis:ti,ab) AND ('herpesvirus 2, human'/exp OR 'herpes simplex virus type 2':ti,ab OR 'herpes simplex virus 2':ti,ab OR 'hsv 2':ti,ab OR 'genital herpes':ti,ab)AND ('symptom' OR 'symptoms')
Cochrane	([mh Microbiota] OR [mh "vaginosis, bacterial"] OR Microbiota:ti,ab OR microbiome:ti,ab OR ("vaginal" NEXT microbio*):ti,ab OR ("bacterial" NEXT vagin*):ti,ab OR ("female reproductive tract" NEXT microbio*):ti,ab OR "vaginal dysbiosis":ti,ab OR vaginitis:ti,ab) AND ([mh "herpesvirus 2, human"] OR "herpes simplex virus type 2":ti,ab OR "herpes simplex virus 2":ti,ab OR HSV-2:ti,ab OR "genital herpes":ti,ab) AND "symptom*"

Web of Science	(Microbiota:ti,ab OR microbiome:ti,ab OR 'vaginal microbio*':ti,ab OR 'bacterial vagin*':ti,ab OR 'female reproductive tract microbio*':ti,ab OR 'vaginal dysbiosis':ti,ab OR vaginitis:ti,ab) AND ('herpesvirus 2, human'/exp OR 'herpes simplex virus type 2':ti,ab OR 'herpes simplex virus 2':ti,ab OR HSV-2:ti,ab OR 'genital herpes':ti,ab) AND all=symptom*
Google Scholar	allintitle: Herpes simplex virus 2 AND ("bacterial vaginosis" OR "vaginal microbiome" OR "vaginal dysbiosis" OR "Vaginal microbiota" OR "female reproductive tract microbiome") AND ("symptom" OR "symptoms")

Supplementary Table S7. Quality Assessment of Studies Included in Primary Outcome Using JBI Critical Appraisal tool for Cohort Studies.

Study (Year)	Were the two groups similar and recruited from the same population?	Were the exposures measured similarly to assign people to both exposed and unexposed groups?	Was the exposure measured in a valid and reliable way?	Were confounding factors identified?	Were strategies to deal with confounding factors stated?	Were the groups/participants free of the outcome at the start of the study?	Were the outcomes measured in a valid and reliable way?	Was the follow up time reported and sufficient to be long enough for outcomes to occur?	Was a follow up complete, and if not, were the reasons to loss to follow up described and explored?	Were stategies to address incomplete follow up utilized?	Was appropriate analysis used?	Overall Appraisal
Cherpes et al (2003)	Y	Y	Y	Y	Y	Y	Y	Y	Y	N/A	Y	Include
Chohan et al (2009)	Y	Y	Y	Y	Y	Y	Y	Y	Y	N/A	Y	Include
Gallo et al (2008)	Y	Y	Y	Y	Y	Y	Y	Y	Y	N/A	Y	Include
Gottleib (2003)	Y	Y	Y	Y	Y	Y	Y	Y	Unclear	No	Y	Include

Supplementary Table S8. Quality Assessment of Studies Included in Secondary Outcome Using JBI Critical Appraisal tool for Cross-Sectional Studies.

Author (Year)	Were the criteria in the sample clearly defined?	Were the study subjects and the setting described in detail?	Was the exposure measured in a valid and reliable way?	Were objective, standard critera used for measurement of the condition?	Were confounding factors identified?	Were strategies to deal with confounding factors stated?	Were the outcomes measured in a valid and reliable way?	Was appropriate statistical analysis used?	Overall appraisal
Baisley et al (2009)	Y	Y	Y	Y	Y	Y	Y	Y	Include
Cherpes et al (2005)	Y	Y	Y	Y	Y	Y	Y	Y	Include

Supplementary Table S9: Summary of Studies of the Association between BV and HSV-2 Viral Shedding

Authors	Year	Country	Study Type	Aim	Study Population	Number of Participants	Result	Measure of Effect	Estimate (95% CI)	Risk of Bias
Baisley et al.	2009	Tanzania	Cross-Sectional Study	To determine prevelance of, and risk factors for, bacterial vaingosis among herpes simplex virus 2 seropositive women at enrollment into a randomized placebo-controlled trial of HSV-suppressive treatment	HSV-2 seropositive, non-pregnant, non-breastfeeding women (16-35 y/o) with no history of epilepsy working in bars, guesthouses, and similar facilities in Lake Victoria region of Tanzania.	1304	HSV-2 shedding resulted in a decreased odds of having BV	OR	0.93; (0.60 to 1.46)	Low
Cherpes et al.	2005	USA	Prospective Cohort Study	To investigate the effects of vaginal coinfections and hormonal contraceptive use on genital tract shedding in women.	Asymptomatic, HSV-2 seropositive, non-pregnant, women (18-30 y/o) who were not using anti-viral agents.	330	BV was identified as an independent risk factor for HSV-2 shedding	OR	2.3;(1.3-4.0)	Low

Supplementary Table S10: Funnel Plot Results for both sets of meta-analyses. Each study's observed outcome (HR) against its standard error are compared to one another.

Funnel Plot Results			
**Adjusted or Unadjusted P values**	**Study Name**	**Standard Error**	**Observed Outcome (HR)**
Adjusted	Cherpes et al, 2003	1.2	2.1
	Chohan et al, 2009	0.68	1.6
	Gallo et al, 2008	1.3	2.4
	Gottlieb et al, 2004	0.81	1.9
Unadjusted	Cherpes et al, 2003	1.1	2.3
	Chohan et al, 2009	0.54	1.7
	Gallo et al, 2008	1.3	2.4
	Gottlieb et al, 2004	0.74	2
